# Palatal rugae morphology is associated with variation in tooth number

**DOI:** 10.1038/s41598-020-76240-w

**Published:** 2020-11-05

**Authors:** Jessica Armstrong, Jadbinder Seehra, Manoharan Andiappan, Allan G. Jones, Spyridon N. Papageorgiou, Martyn T. Cobourne

**Affiliations:** 1 Department of Orthodontics, Faculty of Dentistry, Oral & Craniofacial Sciences, Centre for Craniofacial Development & Regeneration, King’s College London, Floor 27, Guy’s Hospital, London, SE1 9RT UK; 2 Department of Orthodontics, Kingston Hospital NHS Foundation Trust, Kingston, UK; 3 Faculty of Dentistry, Oral & Craniofacial Sciences, Centre for Host-Microbiome Interactions, King’s College London, London, UK; 4 Clinic of Orthodontics and Pediatric Dentistry, Center of Dental Medicine, University of Zurich, Zurich, Switzerland

**Keywords:** Genetic association study, Anatomy, Preclinical research

## Abstract

This observational study compared palatal rugae morphology in adolescent subjects with normal tooth number and tooth agenesis. Maxillary dental study casts were used to compare rugae number, length and shape. Each study group contained 60 subjects (30 females and 30 males) mean age 13.4 (SD, 1.55) in control and 13.56 (SD, 1.54) years in tooth agenesis groups (*p* = 0.576). Mean number of missing tooth units in the tooth agenesis group was 2.1. Mean number of primary rugae in the whole sample was 4.35 (SD, 0.98) on the right and 4.33 (SD, 0.92) on the left with no significant differences (*p* = 0.236 and *p* = 0.404, respectively). However, the number of secondary rugae on the left (*p* = 0.006) and fragmentary rugae on the right (*p* = 0.004) was significantly increased in the tooth agenesis group. The shape of left primary rugae 2 and 3 also differed between groups, tending towards a wavy pattern in the control group and curved in the tooth agenesis group (*p* = 0.012 and *p* = 0.004, respectively). In addition, primary rugae 3 was more convergent (*p* = 0.008) whilst left primary rugae 3 and 5 were orientated in an antero-posterior direction (*p* = 0.04 for both rugae) in the tooth agenesis group. Subgroup analysis also identified significant associations between patterns of tooth agenesis and rugae number, in addition to shape of primary rugae. The identification of significant differences in rugae pattern between subjects with normal tooth number and agenesis suggests potential commonality in signal pathway disruption during establishment of these structures.

## Introduction

The mammalian palatal rugae are a series of conserved transverse ridges situated on the anterior hard palate that extend laterally from the incisive papilla and median raphe with a periodicity and morphology that contribute to a unique pattern^1^. This pattern demonstrates generic species-specific traits but also within-species individuality; in humans, there are between 2 and 7 rugae of variable morphology situated on each side of the midline^2^, whilst mice usually have 3 continuous antemolar and 5 bifurcated intermolar rugae^3^. The palatal rugae have been ascribed multiple possible functions, potentially acting as an aid to suckling in the newborn and later, during the mastication of solid food. The rugae are well vascularized and innervated through the presence of Merkel cell mechanoreceptors that can respond to touch and pressure^4,5^ and are therefore also likely to mediate sensory perception within the oral cavity throughout life. The gross anatomy of human palatal rugae has been studied using a variety of classification systems^6–10^ and they demonstrate good reproducibility and stability over the short-term^11,12^. These observations have led to the use of rugae pattern in forensic medicine during the identification of individuals from dental casts post-mortem^13–16^.

Periodic patterning is a common anatomical motif in multiple species and is observed in many regions, including the distribution of hair follicles, feathers and skin pigmentation patterns^17–20^. An established molecular model of periodic pattern formation is the Alan Turing two-component reaction–diffusion mechanism^21^, based upon the interaction of a diffusible short-range activator (X) and long-range inhibitor molecule (Y), where X is able to induce both its own production and that of Y; whilst Y in turn, inhibits X; with variation in rates of diffusion between X and Y leading to initial instability of the system and then reorganization into stable periodic patterns^22^. In the mouse embryo, the rugae appear at regions of growth within the palatal shelves, starting with the most posterior (number 8), followed by the most anterior (1 and 2) and then filling in between rugae 8 and the last-formed rugae in the order 3–7^23–25^. There is now some evidence that a reaction–diffusion mechanism is responsible for this initial patterning, with Fibroblast Growth Factor (FGF) signalling postulated to act as the activator X and the secreted morphogen Sonic Hedgehog (SHH) as the inhibitor Y^23^. However, other molecules also influence rugae development within this system, including members of the Bone Morphogenetic Protein (BMP) and WNT signalling families^26,27^, and the rugae morphology of a significant number of mouse mutants have now been described^28^.

There is significant commonality in the molecular mechanisms that regulate formation of many embryonic structures and a host of signaling molecules, receptors and transcription factors are expressed in multiple regions of the developing craniofacial complex^29–33^. The palatal rugae therefore represent an excellent model to further investigate this commonality^28^. In recent years, a number of gene mutations have been identified in association with human forms of tooth agenesis, including *MSX1*^34^, *PAX9*^35^*, WNT10A*^36^ and *AXIN2*^37^. Given that many of these genes and components within the associated signal pathways are also expressed in the rugae^23,24,38^, it can be hypothesised that variation in tooth number might be related to alterations in rugae pattern through disruption of common genetic pathways. There is some preliminary evidence for this, including findings of altered rugae patterns in families with sporadic hypodontia associated with variation in the interferon regulatory factor 6 (*IRF6*) gene^8^ and individuals with variation in *WNT3A* and *WNT11*^39^. More recently, we have demonstrated a significant association between rugae pattern variation and oligodontia in a pilot study of human subjects^40^. In the present study we have compared rugae patterns in a larger cohort of non-syndromic adolescent subjects diagnosed with agenesis of one or more permanent teeth with those demonstrating normal tooth number.

## Methods

### Subject recruitment

All methods in this investigation were undertaken in accordance with the relevant guidelines and regulations relating to research involving patient samples obtained from the United Kingdom National Health Service as regulated by the Health Research Authority, Department of Research and Development at Kingston Hospital NHS Trust and King’s College London. The Health Research Authority NRES Committee South East deemed that the nature of this study meant that it did not require formal review by them. The experimental protocols were approved by the Department of Research and Development, Kingston Hospital NHS Trust, United Kingdom (Project Number: NIRAS025). All subject records used in this investigation formed part of the normal records obtained prior to orthodontic treatment. Informed consent was obtained from all subjects, or if subjects were below the age of 18 years, their parent and/or legal guardian. Subjects with tooth agenesis were identified from a database held in the Orthodontic Department at Kingston Hospital. Unaffected control subjects were identified from those attending for routine orthodontic treatment. Criteria for inclusion in the study were: (1) Caucasian; (2) under 18-years of age; (3) no other genetic relations involved in the study; (4) complete records available prior to any orthodontic treatment; (5) no history of any previous orthodontic treatment interventions; (6) good oral hygiene with no evidence of gingival or palatal inflammation from the clinical records; (7) no history of cleft lip/palate, oro-facial syndromes, pathology, trauma or surgery to the maxillary region; and (8) no significant facial asymmetry or jaw discrepancies. Subjects in the tooth agenesis-group had one or more permanent teeth (excluding third molars) developmentally absent; whilst those in the unaffected control-group had all permanent teeth (excluding third molars) present. The presence and developmental absence of teeth was diagnosed following clinical history and examination, supplemented with panoramic radiography, taken as part of the routine orthodontic care pathway for these subjects. No genomic data was obtained from either subject sample.

### Sample size calculation

Sample size calculation was based upon the findings of a previous pilot study investigating palatine rugae pattern and oligodontia, which found statistically significant pattern variation associated with left rugae 2 and 3 in subjects with oligodontia^40^. Adopting the same experimental proportions of 66% and 38% for curved primary rugae 2 and 3 (and the reference proportions for non-curved rugae of 28% and 16%, respectively) a total of 52 and 116 patients would be needed to identify an existing difference between ruga 2 and ruga 3, respectively (with a chi-square test alpha = 5%, beta = 20% and power of 80%). In order to satisfy both outcomes, an overall sample of 116 was used, which was rounded up to 120 patients overall (60 in each group). All sample size calculations were carried out in Stata SE 14.2 (StataCorp, College Station, Texas, USA).

### Data collection

**Figure 1:**
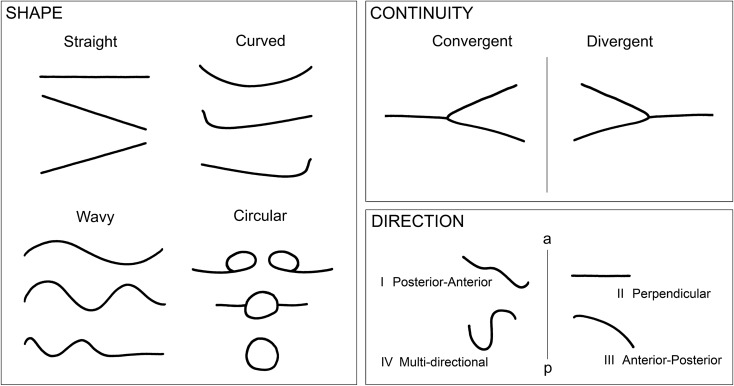
Morphological classification of primary rugae. Primary rugae were classified according to SHAPE, CONTINUITY and DIRECTION. SHAPE was classified as Straight (a straight line from origin to termination), Curved (a simple gently curving crescent shape), Wavy (a serpentine shape or the presence of any curve at the origin or termination of a curved rugae) and Circular (a definite continuous ring). CONTINUITY between rugae was classified as Convergent (a split origin in the midline and converging laterally), Divergent (a single origin in the midline and diverging laterally or Distinct (with no unification and as shown in SHAPE). DIRECTION was classified as Type I–IV according to the relationship between the midline origin and lateral termination in relation to the anterior–posterior axis of the palate midline. Type I (Posterior–Anterior) have an origin situated posterior to the termination; Type II (Perpendicular) have an origin and termination in the same plane; Type III (Anterior–Posterior) have an origin situated anterior to the termination and Type IV are Multi-directional. Vertical lines represent palatal midline. (a) anterior; (p) posterior.

High-quality pre-treatment dental stone casts (Model Stone White Orthodontic Stone, ISO Type 3, Whipmix, USA) of the maxillary arch derived from alginate impressions and including the hard palate were available for all subjects. All rugae were analysed and recorded separately on the right and left side of the palate vault. Briefly, rugae were outlined on the cast using a sharp 6H pencil under illuminated magnification. The total length of each ruga from origin to termination (in mm) was measured using digital callipers accurate to within 0.05 mm (ISO 9001, 150 mm electronic calliper, Tesa Technology, Renens, Switzerland). Rugae were classified as primary, secondary and fragmentary based upon their length (primary = 5–10 mm; secondary = 3–4 mm; fragmentary < 3 mm)^7^. Primary rugae were further analysed according to shape, continuity and direction (Fig. 1). Shape was classified as straight, curved, wavy or circular. Straight rugae run in a straight line from origin to termination; curved rugae have a simple crescent shape curving gently; wavy rugae are serpentine (the presence of any curve at the origin or termination of a curved rugae classifies it as wavy); circular rugae have a definite continuous ring. Continuity between rugae was classified as convergent, divergent or distinct. Convergent rugae have split origins in the midline and converge laterally: divergent rugae have a single origin in the midline and diverge laterally; distinct rugae have no unification. Direction was classified as Type I–IV according to the relationship between midline origin and lateral termination in relation to the anterior–posterior axis of the palate midline. Type I (Posterior–Anterior) have an origin situated posterior to the termination; Type II (Perpendicular) have an origin and termination in the same plane; Type III (Anterior–Posterior) have an origin situated anterior to the termination and Type IV are Multi-directional.

All visible rugae were included in the analysis. All rugae classification and measurement was carried out by the same observer (JA). Ten records were re-evaluated one week apart to assess intra-observer reliability. For rugae length, 128 linear measurements taken and repeated one week later to give overall means of 7.3521 and 7.3073 mm with a correlation of 0.997, indicating no significant difference between repeated measurements.

### Statistical analysis

After checking for normality visually and with the Shapiro–Wilk test, descriptive statistics were calculated including means with Standard Deviations (SD) (or medians with Interquartile Ranges [IQR] for non-normally distributed continuous variables) and absolute/relative frequencies. Independent Student’s *t *test (or Mann–Whitney U test) and Fisher’s exact tests were used to check for differences between tooth agenesis and control groups. For palatal rugae morphology, Cohen’s kappa (two-sided test; design-based formula) was used to calculate agreement between classifications made two weeks apart. A two-sided *p* value of 0.05 was considered statistically significant.

We also undertook subgroup analysis of rugae pattern in relation to commonly described patterns of non-syndromic tooth agenesis. In particular, we subdivided subjects into subgroups (SG1-3) of individuals with absent premolar teeth (*MSX1, WNT10A*) (SG1), permanent maxillary lateral incisor teeth (*EDA, EDAR*) (SG2) and those with tooth agenesis patterns involving absent permanent molar teeth (*PAX9*, *AXIN2*) (SG3)^41^.

No multiplicity correction (such as Bonferroni) was undertaken in the analyses because it was deemed to be unnecessary. The multiple *p* values pertain to different outcomes assessed with independent tests, the results of all of which, are transparently reported and in a hierarchical order^42^.

## Results

**Figure 2:**
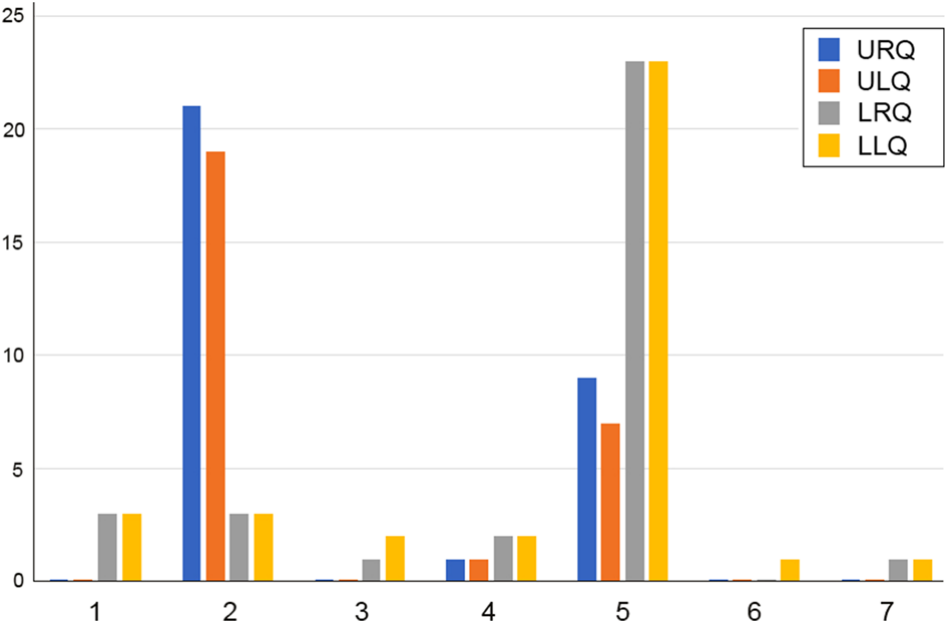
Distribution of missing teeth in the tooth agenesis sample by dental quadrant. The *x* axis represents each tooth in the quadrant from: (1) central incisor; (2) lateral incisor; (3) canine; (4) first premolar; (5) second premolar; (6) first molar and (7) second molar. The *y* axis represents the total number of teeth missing in the tooth agenesis sample. upper right dental quadrant (URQ); upper left dental quadrant (ULQ); lower right dental quadrant (LRQ); lower left dental quadrant (LLQ).

A total sample of 120 subjects were investigated (60 males and 60 females equally distributed between groups) with a mean age of 13.49 (SD, 1.54) years. The control group consisted of 60 subjects (50% female) with a mean age of 13.41 (SD, 1.55) years, whilst the tooth agenesis-group was composed of 60 subjects (50% female) with a mean age of 13.57 (SD, 1.54) years. There were no significant differences in age between control and tooth agenesis groups (*p* = 0.58). Median number of missing teeth (excluding third molars) in the tooth agenesis-group was 2 teeth (IQR 1–3; range 1–6). The distribution of missing teeth in the tooth agenesis group is shown in Fig. 2. It can be seen that mandibular second premolars were the most commonly absent tooth followed by maxillary lateral incisors and maxillary second premolars. The raw data file is included as Supplement File 1.

The mean number of primary rugae in the total sample was 4.35 (SD, 0.98) on the left and 4.33 (SD, 0.93) on the right with no significant differences between groups (Table 1). However, the numbers of secondary rugae on the left (*p* = 0.006) and fragmentary rugae on the right (*p* = 0.004) were significantly increased in the tooth agenesis-group compared to the control-group (Table 1).

**Table 1:** Average number of rugae.

Rugae classification	Experimental group	Statistic	Average	*p* value^a^
Primary rugae right	Control-group	Mean (SD)	4.22 (0.78)	0.17^a^
	Tooth agenesis-group	Mean (SD)	4.45 (1.05)	
Primary rugae left	Control-group	Mean (SD)	4.43 (0.98)	0.36^a^
	Tooth agenesis-group	Mean (SD)	4.27 (0.99)	
Secondary rugae right	Control-group	Median (IQR)	1.00 (0, 2.00)	0.29^b^
	Tooth agenesis-group	Median (IQR)	1.00 (0, 2.00)	
Secondary rugae left	Control-group	Median (IQR)	0 (0, 1.00)	**0.006**^b^
	Tooth agenesis-group	Median (IQR)	0 (0, 1.00)	
Fragmentary rugae right	Control-group	Median (IQR)	0 (0, 0)	**0.004**^b^
	Tooth agenesis-group	Median (IQR)	0 (0, 1.00)	
Fragmentary rugae left	Control-group	Median (IQR)	0 (0, 0)	0.09^b^
	Tooth agenesis-group	Median (IQR)	0 (0, 1.00)	

^a^Student’s t-test.

^b^Independent samples Mann–Whitney U Test.

Significance indicated in bold.

IQR, interquartile range; SD, standard deviation.

Mean rugae lengths in the two groups are shown in Table 2. There were no significant differences in mean length of primary, secondary or fragmentary rugae between groups.

**Table 2:** Mean rugae length.

Rugae number	Experimental group	n	Statistic	Average	*p* value
**Primary rugae right**					
1	Control-group	60	Mean (SD)	8.78 (1.56)	0.33^a^
	Tooth agenesis-group	60	Mean (SD)	8.48 (1.80)	
2	Control-group	60	Median (IQR)	8.83 (8.03, 10.87)	0.66^b^
	Tooth agenesis-group	60	Median (IQR)	9.28 (7.30, 10.3)	
3	Control-group	60	Median (IQR)	9.69 (7.87, 11.94)	0.36^b^
	Tooth agenesis-group	59	Median (IQR)	8.93 (7.09, 11.83)	
4	Control-group	51	Median (IQR)	8.38 (6.68, 11.49)	0.16^b^
	Tooth agenesis-group	52	Median (IQR)	9.27 (7.62, 12.22)	
5	Control-group	18	Median (IQR)	7.11 (6.39, 9.10)	0.80^b^
	Tooth agenesis-group	24	Median (IQR)	7.16 (6.22, 8.80)	
6	Control-group	4	Mean (SD)	6.89 (1.15)	0.93^a^
	Tooth agenesis-group	8	Mean (SD)	6.98 (1.98)	
7	Control-group	0	–	–	–
	Tooth agenesis-group	3	Median (IQR)	5.25 (5.09, 9.21)	
**Primary rugae left**					
1	Control-group	60	Mean (SD)	9.15 (1.49)	0.91^a^
	Tooth agenesis-group	60	Mean (SD)	9.12 (1.69)	
2	Control-group	60	Median (IQR)	9.06 (7.38, 10.18)	0.77^b^
	Tooth agenesis-group	60	Median (IQR)	9.34 (6.94, 10.71)	
3	Control-group	59	Median (IQR)	9.08 (7.14, 11.52)	0.40^b^
	Tooth agenesis-group	59	Median (IQR)	9.96 (8.14, 10.82)	
4	Control-group	52	Median (IQR)	9.74 (6.95, 11.78)	0.44^b^
	Tooth agenesis-group	46	Median (IQR)	9.37 (6.34, 11.91)	
5	Control-group	26	Median (IQR)	8.04 (7.12, 9.31)	0.09^b^
	Tooth agenesis-group	24	Median (IQR)	9.40 (7.47, 11.42)	
6	Control-group	7	Median (IQR)	6.63 (6.30, 10.39)	0.85^b^
	Tooth agenesis-group	7	Median (IQR)	6.86 (5.72, 12.40)	
7	Control-group	2	Median (IQR)	5.45 (5.19, 5.70)	
	Tooth agenesis-group	0	–	–	
**Secondary rugae right**					
1	Control-group	37	Median (IQR)	3.97 (3.49, 4.51)	0.35^b^
	Tooth agenesis-group	40	Median (IQR)	4.10 (3.53, 4.66)	
2	Control-group	16	Mean (SD)	4.05 (0.59)	0.61^a^
	Tooth agenesis-group	20	Mean (SD)	4.14 (0.47)	
3	Control-group	1	Median (IQR)	3.37 (–)	–
	Tooth agenesis-group	11	Mean (SD)	3.91 (0.67)	
4	Control-group	0	–	–	–
	Tooth agenesis-group	2	Median (IQR)	4.41 (3.86, 4.96)	
**Secondary rugae left**					
1	Control-group	25	Median (IQR)	3.93 (3.59, 4.12)	0.44^b^
	Tooth agenesis-group	38	Median (IQR)	3.97 (3.50, 4.66)	
2	Control-group	10	Mean (SD)	4.15 (0.43)	0.20^a^
	Tooth agenesis-group	17	Mean (SD)	3.89 (0.52)	
3	Control-group	2	Median (IQR)	3.90 (3.14, 4.65)	0.86^a^
	Tooth agenesis-group	6	Mean (SD)	4.00 (0.58)	
4	Control-group	0	–	–	–
	Tooth agenesis-group	1	Median (IQR)	4.28 (–)	
**Fragmentary rugae right**					
1	Control-group	11	Mean (SD)	2.40 (0.27)	0.29^a^
	Tooth agenesis-group	27	Mean (SD)	2.25 (0.43)	
2	Control-group	3	Mean (SD)	2.44 (0.68)	0.97^a^
	Tooth agenesis-group	8	Mean (SD)	2.42 (0.43)	
3	Control-group	2	Median (IQR)	2.19 (1.83, 2.54)	–
	Tooth agenesis-group	1	Median (IQR)	1.95 (–)	
**Fragmentary rugae left**					
1	Control-group	9	Median (IQR)	2.53 (2.08, 2.75)	0.71^b^
	Tooth agenesis-group	17	Median (IQR)	2.52 (2.29, 2.72)	
2	Control-group	3	Mean (SD)	2.39 (0.28)	0.25^a^
	Tooth agenesis-group	5	Mean (SD)	1.92 (1.19)	

^a^Student’s *t* test.

^b^Independent samples Mann–Whitney U Test.

Significance indicated in bold.

IQR, interquartile range; SD, standard deviation.

The distribution of primary rugae shape within the two groups is shown in Table 3. The shape of primary rugae 2 on the left was significantly different between groups (*p* = 0.01). In the control-group, this rugae was wavier (50% compared to 27% in the agenesis-group); whilst in the tooth agenesis-group it was more curved (70% compared to 43% in the control-group). In addition, the shape of primary rugae 3 and primary rugae 4 on the right was significantly different between groups (*p* = 0.004 and *p* = 0.02, respectively). Specifically, in the control-group both rugae 3 and 4 were wavier, whilst in the tooth agenesis-group they were more curved.

**Table 3:** Primary rugae shape.

Rugae number	Experimental group	Primary rugae shape				*p* value^a^
		Straight	Curve	Wavy	Circular	
**Primary rugae right**						
1	Control-group	3 (5%)	42 (70%)	12 (20%)	3 (5%)	0.32
	Tooth agenesis-group	6 (10%)	46 (77%)	6 (10%)	2 (3%)	
2	Control-group	1 (2%)	32 (53%)	27 (45%)	0 (0%)	0.58
	Tooth agenesis-group	1 (2%)	34 (57%)	23 (38%)	2 (3%)	
3	Control-group	0 (0%)	17 (28%)	43 (72%)	0 (0%)	**0.004**
	Tooth agenesis-group	4 (7%)	28 (47%)	27 (46%)	0 (0%)	
4	Control-group	1 (2%)	12 (24%)	38 (75%)	0 (0%)	**0.02**
	Tooth agenesis-group	0 (0%)	24 (46%)	28 (54%)	0 (0%)	
5	Control-group	1 (6%)	8 (44%)	9 (50%)	0 (0%)	0.28
	Tooth agenesis-group	0 (0%)	15 (63%)	9 (38%)	0 (0%)	
6	Control-group	0 (0%)	1 (25%)	3 (75%)	0 (0%)	0.42
	Tooth agenesis-group	0 (0%)	4 (50%)	4 (50%)	0 (0%)	
7	Control-group	0 (0%)	0 (0%)	0 (0%)	0 (0%)	–
	Tooth agenesis-group	0 (0%)	2 (67%)	1 (33%)	0 (0%)	
**Primary rugae left**						
1	Control-group	1 (2%)	44 (73%)	12 (20%)	3 (5%)	0.49
	Tooth agenesis-group	5 (8%)	40 (67%)	12 (20%)	3 (5%)	
2	Control-group	3 (5%)	26 (43%)	30 (50%)	1 (2%)	**0.01**
	Tooth agenesis-group	2 (3%)	42 (70%)	16 (27%)	0 (0%)	
3	Control-group	3 (5%)	17 (28%)	40 (67%)	0 (0%)	0.60
	Tooth agenesis-group	2 (3%)	22 (37%)	35 (59%)	0 (0%)	
4	Control-group	0 (0%)	18 (35%)	34 (65%)	0 (0%)	0.40
	Tooth agenesis-group	0 (0%)	18 (39%)	28 (61%)	0 (0%)	
5	Control-group	0 (0%)	8 (31%)	18 (69%)	0 (0%)	0.25
	Tooth agenesis-group	0 (0%)	11 (46%)	12 (50%)	1 (4%)	
6	Control-group	0 (0%)	4 (57%)	3 (43%)	0 (0%)	0.50
	Tooth agenesis-group	0 (0%)	3 (43%)	4 (57%)	0 (0%)	
7	Control-group	0 (0%)	2 (100%)	0 (0%)	0 (0%)	–
	Tooth agenesis-group	0 (0%)	0 (0%)	0 (0%)	0 (0%)	

^a^Fishers exact test.

Significance indicated in bold.

Continuity patterns in the two groups are shown in Table 4. There was a significant difference in unification of primary rugae number 3 on the left (*p* = 0.008). Specifically, this rugae was more convergent in the tooth agenesis-group (17%) when compared to the control-group (2%).

**Table 4:** Primary rugae continuity.

Rugae number	Experimental group	Primary rugae continuity			*p* value^a^
		Convergent	Divergent	Neither	
**Primary rugae right**					
1	Control-group	9 (15%)	3 (5%)	48 (80%)	0.77
	Tooth agenesis-group	6 (10%)	3 (5%)	51 (85%)	
2	Control-group	12 (20%)	3 (5%)	45 (75%)	0.86
	Tooth agenesis-group	12 (20%)	5 (8%)	43 (72%)	
3	Control-group	6 (10%)	1 (2%)	53 (88%)	0.30
	Tooth agenesis-group	10 (17%)	3 (5%)	46 (78%)	
4	Control-group	3 (6%)	2 (4%)	46 (90%)	0.78
	Tooth agenesis-group	5 (10%)	1 (2%)	46 (88%)	
5	Control-group	0 (0%)	1 (6%)	17 (94%)	0.68
	Tooth agenesis-group	1 (4%)	0 (0%)	23 (96%)	
6	Control-group	0 (0%)	0 (0%)	4 (100%)	–
	Tooth agenesis-group	0 (0%)	0 (0%)	8 (100%)	
7	Control-group	0 (0%)	0 (0%)	0 (0%)	–
	Tooth agenesis-group	0 (0%)	0 (0%)	3 (100%)	
**Primary rugae left**					
1	Control-group	7 (12%)	3 (5%)	5 (83%)	1.00
	Tooth agenesis-group	7 (12%)	2 (3%)	51 (85%)	
2	Control-group	8 (13%)	3 (5%)	49 (82%)	0.94
	Tooth agenesis-group	10 (17%)	3 (5%)	47 (78%)	
3	Control-group	1 (2%)	1 (2%)	57 (97%)	**0.008**
	Tooth agenesis-group	10 (17%)	1 (2%)	48 (81%)	
4	Control-group	0 (0%)	1 (2%)	51 (98%)	0.08
	Tooth agenesis-group	4 (9%)	2 (2%)	92 (89%)	
5	Control-group	0 (0%)	0 (0%)	26 (100%)	0.23
	Tooth agenesis-group	2 (8%)	0 (0%)	22 (92%)	
6	Control-group	0 (0%)	0 (0%)	7 (100%)	0.50
	Tooth agenesis-group	1 (14%)	0 (0%)	6 (86%)	
7	Control-group	0 (0%)	0 (0%)	2 (100%)	–
	Tooth agenesis-group	0 (0%)	0 (0%)	0 (0%)	

^a^Fisher’s exact test.

Significance indicated in bold.

There were no significant differences in rugae direction pattern between groups (Table 5), except for primary rugae 3 and 5. For both these rugae, their direction was significantly more Type I (posterior-anterior) in the tooth agenesis-group (73% and 71%) compared to the control-control (56% and 42%).

**Table 5:** Primary rugae direction.

Rugae number	Experimental group	Direction of rugae				*p* value^a^
		Type I	Type II	Type III	Type IV	
**Primary rugae right**						
1	Control-group	40 (67%)	2 (3%)	18 (30%)	0 (0%)	0.76
	Tooth agenesis-group	43 (72%)	1 (2%)	16 (27%)	0 (0%)	
2	Control-group	22 (37%)	0 (0%)	38 (63%)	0 (0%)	0.37
	Tooth agenesis-group	18 (30%)	2 (3%)	30 (67%)	0 (0%)	
3	Control-group	19 (32%)	0 (0%)	41 (68%)	0 (0%)	0.57
	Tooth agenesis-group	18 (31%)	2 (3%)	39 (66%)	0 (0%)	
4	Control-group	16 (31%)	0 (0%)	35 (69%)	0 (0%)	0.47
	Tooth agenesis-group	15 (29%)	0 (0%)	37 (71%)	0 (0%)	
5	Control-group	5 (28%)	0 (0%)	13 (72%)	0 (0%)	0.22
	Tooth agenesis-group	11 (44%)	0 (0%)	14 (56%)	0 (0%)	
6	Control-group	2 (50%)	0 (0%)	2 (50%)	0 (0%)	0.24
	Tooth agenesis-group	1 (13%)	0 (0%)	7 (88%)	0 (0%)	
7	Control-group	0 (0%)	0 (0%)	0 (0%)	0 (0%)	–
	Tooth agenesis-group	2 (67%)	0 (0%)	1 (33%)	0 (0%)	
**Primary rugae left**						
1	Control-group	42 (70%)	1 (2%)	16 (27%)	1 (2%)	0.57
	Tooth agenesis-group	48 (80%)	1 (2%)	10 (17%)	1 (2%)	
2	Control-group	33 (55%)	2 (3%)	25 (42%)	0 (0%)	0.17
	Tooth agenesis-group	42 (70%)	1 (2%)	16 (27%)	1 (2%)	
3	Control-group	23 (56%)	0 (0%)	26 (44%)	0 (0%)	**0.04**
	Tooth agenesis-group	43 (73%)	0 (0%)	16 (27%)	0 (0%)	
4	Control-group	30 (58%)	0 (0%)	22 (42%)	0 (0%)	0.22
	Tooth agenesis-group	31 (67%)	0 (0%)	15 (33%)	0 (0%)	
5	Control-group	11 (42%)	0 (0%)	15 (58%)	0 (0%)	**0.04**
	Tooth agenesis-group	17 (71%)	0 (0%)	7 (29%)	0 (0%)	
6	Control-group	4 (57%)	0 (0%)	3 (43%)	0 (0%)	0.56
	Tooth agenesis-group	5 (71%)	1 (14%)	1 (14%)	0 (0%)	
7	Control-group	2 (100%)	0 (0%)	0 (0%)	0 (0%)	
	Tooth agenesis-group	0 (0%)	0 (0%)	0 (0%)	0 (0%)	

^a^Fisher’s exact test.

Significance indicated in bold.

Table 6 gives the agreement between repeated measurement of palatal rugae morphology. Agreement for right rugae was substantial (rugae 1, 2, and 5) or almost perfect (rugae 3, 4, 6, and 7). Agreement for the left rugae again was substantial (rugae 1 and 3) or almost perfect (rugae 2, 4, 5, and 6).

**Table 6:** Agreement for palatal ruga morphology with Kappa.

Ruga	Right			Left		
	n	% Observed agreement (95% CI)	Kappa (95% CI)	n	% Observed agreement (95% CI)	Kappa (95% CI)
1st	19	94.7% (83.7%, 100.0%)	0.64 (− 0.06, 1.00)	19	89.5% (74.3%, 100.0%)	0.73 (0.34, 1.00)
2nd	19	84.2% (66.2%, 100.0%)	0.68 (0.33, 1.00)	19	94.7% (83.7%, 100.0%)	0.89 (0.66, 1.00)
3rd	19	94.7% (83.7%, 100.0%)	0.90 (0.68, 1.00)	19	89.5% (74.3%, 100.0%)	0.79 (0.50, 1.00)
4th	19	94.7% (83.7%, 100.0%)	0.89 (0.66, 1.00)	15	93.3% (79.0%, 100.0%)	0.86 (0.55, 1.00)
5th	8	87.5% (57.9, 100.0%)	0.75 (0.18, 1.00)	10	90.0% (67.4%, 100.0%)	0.83 (0.45, 1.00)
6th	4	100.0% (–)	1.00 (–)	2	100.0% (–)	1.00 (–)
7th	1	100.0% (–)	1.00 (–)	0	–	–

CI, confidence interval.

For the subgroup analysis we identified significant associations between rugae number and patterns of tooth agenesis (Supplement File 2). All three tooth agenesis patterns had more left-sided secondary rugae 2 than controls, whilst specific rugae number deviations from controls were also seen for individual tooth agenesis patterns. In particular, those with premolar agenesis (SG1) had more right-sided fragmentary rugae, upper lateral incisor agenesis (SG2) had fewer left-sided primary rugae and molar agenesis (SG3) had more right-sided secondary and left-sided fragmentary rugae than the control group. There were also differences in terms of rugae shape and tooth agenesis patterns, with premolar agenesis and the upper lateral incisor agenesis patterns having a wavy primary rugae 3 significantly less often than the control group, a finding also seen for premolar agenesis patterns in relation to the primary right rugae 4.

## Discussion

We have further investigated pattern variation in the palatal rugae of human subjects with normal tooth number compared to a group with tooth agenesis. This study represents the largest sample of subjects investigated to date for associations between rugae pattern and tooth number. Although there were no significant differences in the number of primary rugae between groups, significant differences were identified in their shape. Specifically, there was evidence of more curvature unilaterally for primary rugae 2 (left) and 3 (right), more convergence for primary ruga 3 and an antero-posterior direction for left primary rugae 3 and 5 in the tooth-agenesis group. Moreover, the number of secondary rugae (left) and fragmentary rugae (right) was significantly increased in the tooth agenesis group; whilst subgroup analysis identified some significant associations between patterns of tooth agenesis and both number and patterns of rugae.

This observational study builds upon a previous pilot investigation, which identified rugae pattern differences in a smaller sample of subjects with a more severe form of tooth agenesis (oligodontia, associated with a mean number of 8.7 missing teeth, excluding third molars)^40^. Although this investigation used a simpler method of rugae classification, there was a significantly increased frequency of curved rugae seen on the left side in association with tooth agenesis^40^. Moreover, borderline associations have also been identified between variation in *IRF6* and primary rugae size and shape in a group of subjects with sporadic tooth-agenesis^8^. Collectively, these studies suggest potential commonality in the signalling pathways involved in regulating human tooth number and rugae pattern.

In this study we were careful to limit the analysis to a Caucasian sample because there is evidence that rugae pattern does vary between different ethnic groups and populations, and this can include variation in both shape^43,44^ and number^6,45^, although not all studies have found significant differences^46^. Similarly, we ensured equal numbers of males and females in each experimental group because gender-based differences in rugae number and pattern have been reported within some populations^47–50^; although again, other studies have found no significant gender-related differences^6,51–53^. Longitudinal data in non-treated subjects has shown the rugae to be stable in their antero-posterior relationships, length and inter-rugae distances, with only very minimal changes during development^54^ making them amenable to the type of single time-point comparison in two similarly aged groups that was performed in the present investigation^11,12^.

It has been demonstrated in the mouse embryo that an activator-inhibitor system dependent upon FGF and SHH signalling, respectively is responsible for the periodic generation of rugae within specific growth zones in the developing palate^23^. In the mouse, loss-of-function associated with *Fgf10* or *Fgfr2* results in an absence of palatal rugae, consistent with a role for FGF signalling as an activator within this system^25,55^. Moreover, loss of the Sprouty1/Sprouty2 intra-cellular FGF anatagonists results in highly disorganised rugae patterns, including broader and ectopic rugae distributed in a widespread manner across the palate^23,56^; whilst conditional loss of *Shh* in the oral epithelium or pharmacological inhibition in palatal shelf explants also results in the formation of highly disorganised and ectopic rugae^23,57^. Together, these data suggest that much like Sprouty, SHH acts as an inhibitor within this system. However, it is clear that other major signalling pathways are also involved in modifying this patterning process, including signalling between rugae epithelium and mesenchyme within the developing palatal shelves. These include WNT signalling, with ablation of signal in the oral epithelium preventing rugae formation^26^ and BMP signalling^24^. Indeed, loss-of-function mutations in *Sostdc1*, which encodes a secreted BMP anatagonist and WNT modulator, results in a failure of fusion associated with anterior ruga 4 and disorganized rugae morphology in the mouse^25,38^. Although human rugae morphology is more complex than that in the mouse, the fundamental mechanisms are likely to be conserved at the molecular level^28^. It is clear that further investigation would ideally involve a larger cohort of subjects with more detailed phenotypic data. In particular, having genomic data would allow further delineation of the underlying genetic mechanisms and associations that are potentially involved in human rugae development and build upon what is currently a sparse knowledge base^8^, albeit one with significant potential for further research.

In addition to the lack of genomic data, there are a number of other potential limitations to this study. The prevalence and patterns of tooth agenesis seen in our sample were consistent with those reported from epidemiological studies investigating non-syndromic forms of selective tooth agenesis^58^. It can be seen that mandibular second premolars were the most commonly absent tooth followed by maxillary lateral incisors and maxillary second premolars. We did undertake a limited subgroup analysis of rugae number and pattern in association with common patterns of tooth agenesis described in the literature in association with single gene mutations^41^ and identified by phenotype within the sample. We found some associations between patterns of tooth agenesis and rugae number; however, without definitive mutational analysis it is difficult to draw any significant conclusions from these data. Moreover, we cannot exclude a syndromic basis for any cases of tooth agenesis within our sample, particularly in the absence of genomic data and it is well established that missing teeth can represent a relatively common manifestation associated with a number of developmental disorders^59^. This means that potentially, a sample of individuals demonstrating selective tooth agenesis might be quite heterogenous in terms of the developmental origins of their tooth absence. Any subjects diagnosed with underlying medical problems were excluded but it is entirely possible that some were harbouring mutations in potentially important genes and this could have introduced bias into the study. There is also a degree of subjectivity in the identification and classification of rugae, which is reflected in the wide range of classification systems that have been described. However, we used rugae length as the defining arbiter of primary, secondary or tertiary classification^7^ and this was associated with good reproducibility. Moreover, the reproducibility of shape delineation was also excellent, which suggests that the employed method was sufficiently robust.

This observational study compared palatal rugae morphology in 120 adolescent subjects with normal tooth number and selective tooth agenesis. The number of secondary rugae on the left and fragmentary rugae on the right was significantly increased in the tooth agenesis group. The shape of left primary rugae 2 also differed between groups, being more wavy in the controls and curved in the tooth agenesis group. Right primary ruga 3 also differed in shape, being wavy in the control and curved in the tooth agenesis group. Finally, the left primary rugae 3 was additionally more convergent in the agenesis group, while the left primary rugae 3 and 5 had more often an antero-posterior direction in the tooth agenesis group. The identification of significant differences in rugae number and shape between subjects with normal tooth number and agenesis suggests commonly disrupted developmental pathways during establishment of these structures.

## Supplementary information

Supplementary information 1.

Supplementary information 2.
