# Supplementary material for: Palatal rugae morphology is associated with variation in tooth number
Source: Sci Rep. 2020 Nov 5;10:19074. doi: 10.1038/s41598-020-76240-w (PMC7645628; doi:10.1038/s41598-020-76240-w)
Supplement: Supplementary file 2 — Supplementary information 2. [file 41598_2020_76240_MOESM2_ESM.docx]

**Supplement 2 Subgroup analysis for average number of rugae or primary rugae shape and patterns of tooth agenesis**

|  | **Control patients compared to those having agenesis of…** | | |  |
| --- | --- | --- | --- | --- |
| **Rugae classification** | **Premolar agenesis (SG1)** | **Upper lateral incisor agenesis (SG2)** | **Molar agenesis (SG3)** | **Compared results** |
|  | **P value** | **P value** | **P value** |  |
| Primary rugae right | 0.17* | 0.63* | 0.80* |  |
| Primary rugae left | 0.38* | **0.03*** | 0.69* | SG2 has fewer rugae than control (means 3.84 versus 4.43) |
| Secondary rugae  right | 0.48^†^ | 0.76^†^ | **0.03^†^** | SG3 has more rugae than control (medians 3.00 versus 1.00) |
| Secondary rugae  left | **0.001^†^** | **0.04^†^** | **0.02^†^** | SG1-3 have more rugae than control (medians 1.00, 1.00, 2.00 versus 0) |
| Fragmentary rugae right | **0.01^†^** | 0.10 | 0.57 | SG1 has more rugae than control (median 0 in both cases) |
| Fragmentary rugae left | 0.32^†^ | 0.42 | **0.0002^†^** | SG3 has more rugae than control (median 3.00 versus 0) |
|  |  |  |  |  |
| Primary rugae right #1 | 0.15^‡^ | 0.41^‡^ | 1.00^‡^ |  |
| Primary rugae right #2 | 1.00^‡^ | 0.42^‡^ | 0.29^‡^ |  |
| Primary rugae right #3 | **0.01^‡^** | **0.02^‡^** | 0.64^‡^ | Control: 0% straight / 28% curve / 72% wavy  SG1: 4% straight / 54% curve / 42% wavy  SG2: 16% straight / 26% curve / 58% wavy |
| Primary rugae right #4 | **0.02^‡^** | 0.41^‡^ | 1.00^‡^ | Control: 2% straight / 24% curve / 75% wavy SG1: 0% straight / 57% curve / 43% wavy  SG2: 0% straight / 42% curve / 59% wavy |
| Primary rugae right #5 | 1.00^‡^ | 1.00^‡^ | 1.00^‡^ |  |
| Primary rugae right #6 | 0.36^‡^ | 0.80^‡^ | - |  |
| Primary rugae right #7 | - | - | - |  |
|  |  |  |  |  |
| Primary rugae left #1 | 0.20^‡^ | 0.30^‡^ | 1.00^‡^ |  |
| Primary rugae left #2 | 0.38^‡^ | 0.07^‡^ | 0.27^‡^ |  |
| Primary rugae left #3 | 0.76^‡^ | 0.90^‡^ | 1.00^‡^ |  |
| Primary rugae left #4 | 0.20^‡^ | 0.25^‡^ | 0.73^‡^ |  |
| Primary rugae left #5 | 0.40^‡^ | 0.41^‡^ | 0.70^‡^ |  |
| Primary rugae left #6 | 0.35^‡^ | 0.72^‡^ | 0.63^‡^ |  |
| Primary rugae left #7 | - | - | - |  |

SG subgroup

* Student’s t-test

^†^ Independent samples Mann-Whitney U Test

^‡^ Fishers exact test
